# General Paresis of the Insane

**Published:** 1905-11-25

**Authors:** 


### GENERAL PARESIS OF THE INSANE.

A clinical study of some interest is furnished by Dr. Edward Hunt.[1] It is based upon a consideration of sixty cases of general paresis affecting males, observed at the Vanderbilt clinic in New York. For the sake of comparing the features of the disease in different countries the author has included a statistical statement of a large series—some 500 cases—of paretics who came under notice at Moscow.

Of the sixty cases the majority were well-to-do artisans and mechanics; the majority were married, at Moscow 79 per cent., at New York 68 per cent. Most of the cases were well advanced, and the patients ranged in age from twenty years upward. The age incidence in the New York series was as follows:—

| | |
|---|---|
| Age 20-29 | 8.7 per cent. |
| Age 30-39 | 33.3 ,, |
| Age 40-49 | 38.5 ,, |
| Age 50-59 | 15.7 ,, |
| Age 60-69 | 3.5 ,, |

Between the ages of thirty-five and forty-five there occurred 61.4 per cent. of the total number of cases. The Russian figures indicate that in that country the years between the thirty-first and thirty-fifth supply the most cases. The character of the knee-jerk in this disease is illustrated by the following comparative table:—

| | Moscow. | New York. |
|---|---|---|
| Exaggerated | 50.57 per cent. | 53.57 per cent. |
| Diminished | 9.09 ,, | 3.57 ,, |
| Abolished | 19.89 ,, | 30.35 ,, |
| Normal | 12.31 ,, | 1.78 ,, |

As regards the condition of the pupils the comparative table appears as follows:—

| | Moscow. | New York. |
|---|---|---|
| Reaction bad or absent | 82.05 per cent. | 71.15 per cent. |
| Reaction normal | 17.9 ,, | 28.8 ,, |

The tremor commonly associated with the disease was present in 76.8 per cent. of cases. The symptom most frequently complained of was loss of memory; the memory was either very bad or completely lost in some 60 per cent. of the cases, while mental symptoms of some kind or other were rarely absent.

The proportion of instances in which previous syphilis was admitted ranged from 61.5 per cent. at Moscow to 59.3 per cent. at New York, and where it was admitted the interval between the infection and the onset of symptoms of paresis varied from five to eighteen years. The most usual interval, however, was about fifteen years. It will be remembered in this connection that various observers have found a history of previous syphilis in percentages of all cases of general paresis ranging from 70 to 90, and that the most prominent syphilographer, Professor Fournier, goes so far as to call general paresis a parasyphilitic affection.

In concluding his communication the author reconstructs from the data he has dealt with the following composite picture of general paresis. A man of about forty-five, fairly well to do and married, has been perfectly well until a year or fifteen months ago. About that time he began to feel nervous, weak, and restless. Becoming gradually worse, he is at last driven to a physician, who finds in him the Argyll-Robertson pupil, facial and lingual tremor, paretic speech, exaggerated knee-jerks, and a faulty memory. When questioned carefully he admits that some fifteen years before he had a chancre.

[1] Med. Rec., N.Y., September 23, 1905.

